# Supplementary material for: Physical Activity Monitors in Companion Animal Chronic Pain Research—A Review Focused on Osteoarthritis Pain
Source: Animals (Basel). 2025 Jul 10;15(14):2025. doi: 10.3390/ani15142025 (PMC12291673; doi:10.3390/ani15142025)
Supplement: Supplementary file 1 [file animals-15-02025-s001.zip › animals-3712595-Supplementary File S1.pdf]

An overview of the various components to validity in the context of physical activity monitors used in chronic pain research

Content Validity:

Evaluating whether the monitor accurately captures the different types and intensities of physical activity relevant to the study or intended use. For example, if the research question is to measure different levels of activity, then content validity might involve assessing if the monitor adequately differentiates between sedentary, light, moderate, and vigorous activity levels.

Construct Validity:

Examining whether the monitor measures the intended concept of physical activity. This may involve correlating monitor data with other measures of physical activity like self-reported questionnaires or observed behavior, or another 'validated' physical activity monitor.

Discriminant validity is often nested within construct validity, and, in the context of chronic pain research, this refers to whether or not the monitor can be used to discriminate between individuals in the painful state versus non-painful state.

Criterion Validity:

Comparing the activity monitor's data to a well-established, highly accurate method, for example, videographic analysis of movement. Ideally, criterion validity involves testing the monitor in controlled laboratory settings and real-world conditions (e.g. home environment).

Given the differing functionality of different monitors, an individual monitor may be valid for detecting certain types of activity, but not others. The influence of demographic variables and data analysis needs to be taken into consideration when assessing validity. It is important to remember than no one measure of validity is perfect – multiple aspects of validity should be evaluated and related to what is to be measured. Validity can vary depending on the species, population, study design and specific research question.
